# Plasma cortisol cut-off concentrations using the dexamethasone suppression test in patients with adrenal incidentalomas

**DOI:** 10.3389/fendo.2026.1872524

**Published:** 2026-09-04

**Authors:** Nanna Thurmann Jørgensen, Stina Willemoes Borresen, Linda Hilsted, Christina Christoffersen, Kasper Brødbæk, Randi Ugleholdt, Claus Larsen Feltoft, Line Koch Skaanning, Per Medbøe Thorsby, Åse Krogh Rasmussen, Ulla Feldt-Rasmussen, Marianne Klose

**Affiliations:** 1 Department of Nephrology and Endocrinology, Copenhagen University Hospital, Rigshospitalet, Copenhagen, Denmark; 2 Department of Clinical Medicine, Faculty of Health and Medical Sciences, Copenhagen University, Copenhagen, Denmark; 3 Department of Clinical Biochemistry, Copenhagen University Hospital, Rigshospitalet, Copenhagen, Denmark; 4 Department of Biomedical Sciences, Faculty of Health and Medical Sciences, Copenhagen University, Copenhagen, Denmark; 5 Department of Endocrinology and Internal Medicine, Copenhagen University Hospital, Herlev, Copenhagen, Denmark; 6 Department of Biochemistry and Immunology, Vejle Hospital, Vejle, Denmark; 7 Hormone Laboratory, Dep of Medical Biochemistry and Endocrinology and Metabolism Research Group, Oslo University Hospital, Oslo, Norway; 8 Department of Growth and Reproduction, Copenhagen University Hospital, Rigshospitalet, Copenhagen, Denmark

**Keywords:** adrenal incidentaloma, assay performance, diagnostic cut-off value, hypercortisolism, mild autonomous cortisol secretion, overnight dexamethasone suppression test

## Abstract

**Purpose:**

Assessment of cortisol secretion in patients with adrenal incidentalomas remains challenging. We evaluated the guideline-recommended cortisol cut-off value for cortisol suppression (50 nmol/L) following a 1 mg overnight dexamethasone suppression test (DST) against a control-derived threshold and compared patient classification using the two thresholds. Secondary objectives included assessing associations of pre-DST ACTH, post-DST dexamethasone, and comorbidities with post-DST cortisol.

**Methods:**

In this cross-sectional study (NCT07350031), pre- and post-DST plasma samples from patients with adrenal incidentalomas (*N* = 108) and matched controls (*N* = 101) were prospectively collected, and cortisol was analysed using immunoassay (Elecsys^®^Cort II) and LC–MS/MS. Post-DST dexamethasone ≥3 nmol/L defined appropriate dexamethasone exposure. The 97.5th percentile of LC–MS/MS-measured post-DST cortisol in controls defined the control-derived threshold. Associations between pre-DST ACTH, post-DST dexamethasone, and post-DST cortisol were assessed. The performance of pre-DST ACTH for identifying post-DST cortisol >50 nmol/L was evaluated using receiver operating characteristic analysis. Logistic regression examined associations between post-DST cortisol and diabetes, dyslipidaemia, hypertension, and osteoporosis.

**Results:**

The 97.5^th^ percentile of post-DST cortisol in controls was 66 nmol/L. The 50 nmol/L threshold corresponded to the 90^th^ percentile of controls and classified 44% of patients as having hypercortisolism, compared with 26% using the control-derived threshold (66 nmol/L). Pre-DST ACTH showed poor discrimination for post-DST cortisol >50 nmol/L (AUC 0.69). Post-DST cortisol was not associated with dexamethasone concentrations or comorbidities.

**Main conclusions:**

The guideline-recommended post-DST cortisol threshold of 50 nmol/L may be too low, suggesting that higher thresholds warrant further evaluation.

## Introduction

Currently, morning plasma cortisol after a 1 mg overnight dexamethasone suppression test (DST) is recommended as the first-line screening test for hypercortisolism by guidelines from the European Society of Endocrinology and the European Network for the Study of Adrenal Tumours (ESE/ENS@T) (1, 2). A post-DST cortisol concentration <50 nmol/L is considered adequate suppression of the adrenal axis, effectively ruling out hypercortisolaemia. However, both the optimal threshold and the rationale for the 50 nmol/L cut-off value remain unclear and are a matter of debate (1–4). Identification of patients with suppressed pre-DST ACTH, indicating ACTH-independent cortisol hypersecretion, may improve the diagnostic value of the DST (5). Furthermore, variability in dexamethasone metabolism may influence DST interpretation, as fast metabolism can result in low morning dexamethasone concentrations, leading to elevated cortisol concentrations (6–9).

Although liquid chromatography–tandem mass spectrometry (LC–MS/MS) is considered the gold standard for steroid quantification, automated immunoassays remain more commonly used in clinical settings due to the higher operational costs of LC–MS/MS, primarily related to laborious manual work. Contemporary immunoassays have shown improved agreement with LC–MS/MS for cortisol measurement; however, their performance at the low cortisol concentrations following DST remains insufficiently evaluated (10–12).

The primary aim of this study was to evaluate the guideline-recommended post-DST plasma cortisol cut-off value of 50 nmol/L against the upper reference limit (97.5^th^ percentile) in age- and sex-matched controls and to compare the classification of patients with adrenal incidentalomas using the guideline-recommended and control-derived cut-off values.

Secondary aims were to assess the diagnostic performance of ACTH in identifying post-DST cortisol >50 nmol/L and the associations between post-DST cortisol and pre-DST ACTH, post-DST dexamethasone, and common comorbidities associated with hypercortisolism, including diabetes, hypertension, dyslipidaemia, and osteoporosis. Furthermore, the agreement between post-DST plasma cortisol measured by the Elecsys^®^Cort II assay and LC–MS/MS was evaluated.

## Materials and methods

### Study design

This was a cross-sectional study conducted between 2017 and 2024 at the Department of Nephrology and Endocrinology, Copenhagen University Hospital, Rigshospitalet, Denmark, the Department of Clinical Biochemistry, Copenhagen University Hospital, Rigshospitalet, Denmark, and the Department of Endocrinology and Internal Medicine, Copenhagen University Hospital, Herlev, Denmark.

### Participants

Participants were patients with newly diagnosed adrenal incidentalomas and a control group.

#### The patient group

After being diagnosed with an adrenal incidentaloma, the patients were referred to either of the two endocrinology departments mentioned above.

Inclusion criteria were age ≥18 years and a new diagnosis of an adrenal incidentaloma (defined as an adrenal mass >1 cm detected on diagnostic imaging performed for non-related reasons).

Exclusion criteria were active cancer; an adrenal incidentaloma suspected of malignancy; bilateral adrenal incidentalomas; pregnancy or breastfeeding; conditions or medical treatments that interfered with dexamethasone and cortisol metabolism and/or measurements (including any glucocorticoid formulation that could not be paused within the relevant timeframes prior to study visits: systemic glucocorticoid treatment within one month; glucocorticoid injections within three months and other forms of glucocorticoid treatment, e.g., topical or inhaled, within 14 days); severe comorbidities, including alcohol abuse and severe psychiatric disease; chronic kidney disease (defined as an estimated glomerular filtration rate <60 ml/min); and inability to provide written informed consent.

#### The control group

The control group was age- and sex-matched and recruited through social media, online participant recruitment platforms, the hospital website, and public posters in Copenhagen, Denmark. All study visits were performed at the Department of Nephrology and Endocrinology, Copenhagen University Hospital, Rigshospitalet, Denmark.

Inclusion criteria were age ≥18 years and no previous history of conditions involving the adrenal glands. The same exclusion criteria as for the patients were applied. The controls had no history of abdominal imaging, and the study protocol did not include adrenal imaging.

The study was approved by the Regional Scientific Ethical Committee in Copenhagen (ID H-16041718) and was conducted in accordance with the Declaration of Helsinki. All participants received written and verbal information about the study and gave informed consent before enrolment.

### Methods

The participants attended three study visits. The first visit was a screening visit. At the second visit, baseline (pre-DST) blood samples were collected between 0800–0900 hours. Next, the participants were instructed to take 1 mg of dexamethasone (Dexamethasone 0.5 mg, GALENpharma GmbH, Kiel, Germany) on the same day between 2300–0000 hours. The following day, participants were asked about compliance with dexamethasone intake, and blood samples were collected between 0800–0900 hours.

Anthropometrics were obtained using standardised procedures (13). Diagnoses of hypertension, dyslipidaemia, diabetes, and osteoporosis were documented based on the clinical information available at the time of study enrolment. Participants were classified as having these conditions if they had a registered diagnosis and/or were prescribed relevant medication. After study completion, all patients proceeded to the routine outpatient work-up for adrenal incidentalomas. Controls with post-DST cortisol >50 nmol/L were offered a repeat test (**Supplementary Table 1**). Persistently abnormal and clinically relevant results prompted referral to the outpatient clinic.

### Biochemical analyses

Heparinised plasma samples were centrifuged and stored at −80 °C until analysis of cortisol and dexamethasone.

#### Cortisol concentrations

Plasma cortisol was measured in routine batches as part of the daily production at the Department of Clinical Biochemistry, Rigshospitalet, Copenhagen University Hospital using the Cobas Pro e801 module, ECLIA-technology, and Elecsys^®^Cort II (Roche Diagnostics, GmbH). For these measurements, the intermediate precision (inter-assay), expressed as the coefficient of variation (CV%) was <8% over the study period.

Plasma cortisol was also measured by LC–MS/MS at Rigshospitalet after the addition of ^13^C-labelleded intern standard for cortisol (Sigma Aldrich, C-214) using a Waters Xevo TQ-S mass spectrometer (Waters, Milford, Massachusetts, USA). The chromatography was obtained by a Acquity UPLC HSS T3 column (Waters). The inter-assay precision was <7% for all QC levels except the lowest level (17.7 nmol/L), which had a CV of 10%.

#### Dexamethasone concentrations

Plasma dexamethasone was analysed at the Department of Biochemistry and Immunology, Vejle Hospital, Denmark, and quantified using d_3_-dexamethasone as internal standard and purified using an OASIS PRiME HLB plate (Waters). Samples were analysed using a UPLC system with a Waters ACQUITY BEH C18 column (1.7-µm, 100 x 2.2 mm) and eluted with a gradient of 0.1% ammonium hydroxide in water and methanol. Transitions monitored were 393.13 > 355.00, 393.13 > 237.00 and 393.13 > 372.96 for dexamethasone, and 396.13 > 358.00 for d_3_-Dexamethasone. Calibrators were prepared in-house in 50% methanol (MeOH). Data were collected and analysed using MassLynx. The LLOQ was 1.3 nmol/L.

#### Pre-DST ACTH concentrations

At the second visit (pre-DST), EDTA plasma samples were placed on ice and immediately analysed for ACTH concentrations at Rigshospitalet and Herlev Hospital (from 2023) using the Immulite 2000XPI (Siemens Healthcare Diagnostics, USA) from 2016–2019 and the Cobas 8000 (Roche Diagnostics GmbH, Switzerland) from 2020 onward. The maximum allowable CVs were 10% and 12% (at 7.0 and 57.5 pmol/L) and 8% and 12% (at 8.4 and 82 pmol/L), respectively. The lower limits of quantification (LLOQs) were 1.0 and 0.6 pmol/L, respectively, and the highest LLOQ was used in the data analysis.

#### CBG concentrations

At the second visit (pre-DST), serum samples were centrifuged and stored at −80 °C until analysis of CBG concentrations. The analysis was performed at the Hormone Laboratory, Oslo University Hospital, Aker Hospital, Norway using a competitive radioimmunoassay (DIAsource ImmunoAssays, Belgium) with total analytical CVs of 8%, 9%, and 10% at 586, 940, and 1344 nmol/L, respectively. The method was accredited according to NS-EN ISO/IEC 17025:2017 and NS-EN ISO 15189:2022.

### Statistical methods

All statistical analyses were performed using R (R Foundation for Statistical Computing, Vienna, Austria). Continuous variables are presented as means (95% confidence intervals (CIs)) if normally distributed or medians (interquartile ranges) for non-normally distributed data, and categorical variables as numbers (percentages). Comparisons between groups were performed using Student’s t–test or the Mann–Whitney U test for continuous variables, and the chi–square test or Fisher’s exact test for categorical variables, as appropriate.

Post-DST cortisol and pre-DST ACTH concentrations followed log-normal distributions. ACTH concentrations below the LLOQ were substituted with a value equal to half the LLOQ (LLOQ/2) and treated as a numeric values for all analyses.

The lower reference limit for post-DST plasma dexamethasone was defined as the 2.5^th^ percentile in controls and relevant subgroups to allow comparison with previously published studies (6, 8). This threshold was used to define adequate dexamethasone exposure for subsequent analyses.

The 95^th^ and the 97.5^th^ percentiles of the post-DST cortisol distribution in the control group, along with the recommended cut-off value of 50 nmol/L, were used to evaluate the proportion of patients exceeding the respective post-DST cortisol thresholds.

Multivariable logistic regression models were used to assess the associations between log-transformed LC–MS/MS post-DST cortisol and the presence of diabetes, dyslipidaemia, hypertension, and osteoporosis in the patient group (Odds ratios (ORs) with 95% CIs). Each disease outcome was modelled separately, adjusting for potential confounders including age, sex, and body mass index (BMI), with variable selection by backward elimination and Bonferroni-corrected p-values for multiple testing (the significance threshold was divided by the number of tests performed). To evaluate the discriminatory ability of post-DST plasma cortisol for diabetes, hypertension, dyslipidaemia, and osteoporosis, receiver operation characteristic (ROC) curve analyses and corresponding areas under the curve (AUCs) with 95% CI were calculated.

Linear regression analyses were used to assess the association between LC–MS/MS post-DST cortisol concentrations and pre-DST (Visit 2) ACTH, BMI, waist-to-hip ratio (WHR), and waist circumference, adjusting for sex and age (slopes with 95% CIs). Associations with dexamethasone concentrations and age were also investigated.

Agreement between Elecsys^®^Cort II and LC–MS/MS was evaluated using Bland–Altman analysis (mean difference and 95% limits of agreement (LoA)) and Passing–Bablok regression to assess systematic and proportional bias (slope and intercept with 95% CIs).

A p-value <0.05 was considered statistically significant.

## Results

### Study population and characteristics

**Figure 1:**
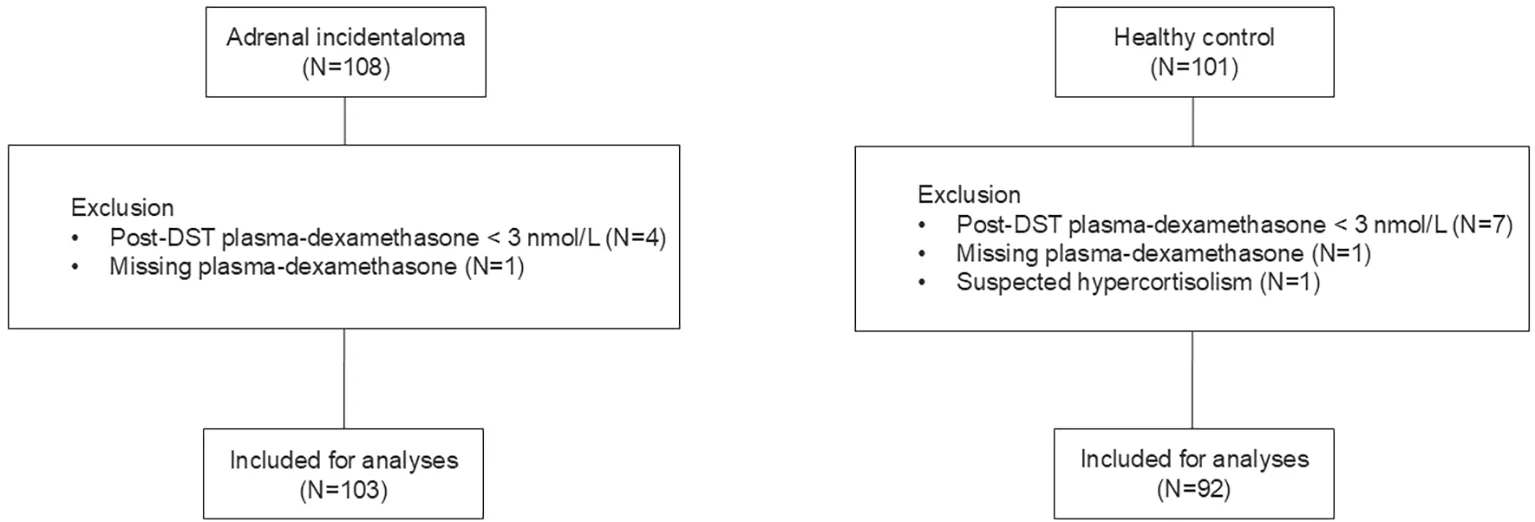
Participant flow. The study included 108 patients with adrenal incidentalomas and 101 controls. Post-DST dexamethasone concentrations were <3.0 nmol/L, corresponding to the 2.5th percentile of the control distribution, in four patients (4%) and six controls (6%), and were missing in one patient and one control. These participants were excluded from subsequent analyses. One additional control was excluded because of markedly elevated post-DST cortisol (>300 nmol/L), suggestive of overt hypercortisolism. The final analyses therefore included 103 patients and 92 controls.

The study included 108 patients with adrenal incidentalomas and 101 controls. After the exclusion of participants with inadequate dexamethasone exposure (for details, see Dexamethasone concentrations) and one control with persistently elevated post-DST cortisol >300 nmol/L, the analysis population comprised 103 patients and 92 controls (**Figure 1**).

Following study completion, three patients underwent adrenal resection, including one with a benign lesion >4 cm and two with clinical and biochemical suspicion of Cushing’s syndrome. A fourth patient underwent biopsy of a hypodense but benign lesion. No diagnoses of adrenocortical carcinoma, phaeochromocytoma, lymphoma, or metastases were made. The diagnosis of Cushing’s syndrome was confirmed in the two patients with clinical and biochemical suspicion of the condition, both of whom remained eligible and were included in the analyses.

**Table 1** shows participant characteristics. Patients with adrenal incidentalomas had a higher BMI and a higher prevalence of diabetes and ≥2 comorbidities than controls. They also had lower pre-DST ACTH concentrations and higher post-DST plasma cortisol concentrations. Other characteristics were comparable between groups.

**Table 1:** Characteristics of patients with adrenal incidentalomas and controls.

Characteristics	Patients (*N* = 103)	Controls (*N* = 92)	*P*
Women, *N* (%)	54 (52)	52 (57)	0.7
Age, years, median (IQR)	63 (55–71)	61 (52–70)	0.2
BMI, kg/m^2^, mean [95% CI]	28.4 [27.4–29.5]	25.9 [24.8–26.9]	<0.001
WHR, mean [95% CI]	0.95 [0.93–0.98]	0.93 [0.92–0.94]	0.07
Pre-DST ACTH, pmol/L, median (IQR)	2.8 (1.7–4.0)	4.6 (3.6–5.8)	<0.001
Post-DST plasma cortisol (LC–MS/MS), nmol/L, median (IQR)	42.5 (33.1–66.7)	30.0 (23.1–40.4)	<0.001
CBG, nmol/L, mean [95% CI]*	978 [945–1011]	956 [930–981]	0.3
Comorbidities, *N* (%)			
Diabetes	15 (15)	1 (1)	<0.001
Dyslipidaemia	42 (41)	28 (30)	0.2
Hypertension	39 (38)	25 (27)	0.2
Osteoporosis	5 (5)	3 (3)	0.7
≥2 comorbidities	33 (32)	14 (15)	0.01
3 comorbidities	9 (9)	1 (1)	0.02

ACTH, adrenocorticotropic hormone; BMI, body mass index; CBG, corticosteroid-binding globulin; DST, 1 mg overnight dexamethasone suppression test; IQR, interquartile range; WHR, waist/hip-ratio.

Results are expressed as mean [95% CI], median (interquartile range), or number (%). Analyses of continuous variables were performed using t–tests for normally distributed variables, and Mann–Whitney U test for non-normally distributed variables. Categorical variables were compared using the chi-square test or Fisher’s exact test, as appropriate.

### Dexamethasone concentrations

Post-DST plasma dexamethasone concentrations were available for 107 patients with adrenal incidentalomas and 98 controls. The 2.5^th^ percentile was approximately 3.0 nmol/L across controls and relevant subgroups (**Table 2**). Six controls (6%) and four patients (4%) had dexamethasone concentrations <3.0 nmol/L and were excluded from the analyses of post-DST cortisol.

**Table 2:** Dexamethasone cut-off values assessed using the 2.5^th^ percentile in controls (N = 98) and relevant subgroups.

Study or subgroup	Dexamethasone cut-off value (nmol/L)*	Controls <cut-off, *N* (%)	Patients <cut-off, *N* (%)
Previous studies			
Ueland et al. (8)	3.3	9 (9)	5 (5)
Genere et al. (6)	2.6	2 (2)	2 (2)
Present study			
Subgroups			
Controls (N = 98)	2.9 [1.8–3.2]	–	–
Controls with post DST cortisol <50 nmol/L (N = 88)	3.0 [2.4–3.4]	–	–
Patients and controls with post-DST cortisol <50 nmol/L (N = 147)	3.0 [2.8–3.8]	–	–
Final locally defined dexamethasone cut-off value	3.0	6 (6)#	4 (4)#

DST; 1-mg overnight dexamethasone suppression test, LC–MS/MS; liquid chromatography–tandem mass spectrometry. Results are presented as the 2.5th percentile (95% CI) of post-DST LC–MS/MS plasma dexamethasone concentrations and the number (N (%)) of participants below the corresponding dexamethasone cut-off values.

*Dexamethasone measurements were available in 98 of 99 controls and 107 of 108 patients.

#Two of the six controls had post-DST cortisol >50 nmol/L. All four patients had post-DST cortisol >50 nmol/L.

All four patients with dexamethasone concentrations <3.0 nmol/L had post-DST cortisol >50 nmol/L (range: 55–190 nmol/L), compared with 42% of patients with dexamethasone concentrations ≥3.0 nmol/L (*P* = 0.04). Among controls with dexamethasone <3.0 nmol/L, two had post-DST cortisol >50 nmol/L. One underwent a repeat DST with adequate dexamethasone exposure, resulting in suppression of post-DST cortisol to 21 nmol/L (**Supplementary Table 1**).

**Figure 2:**
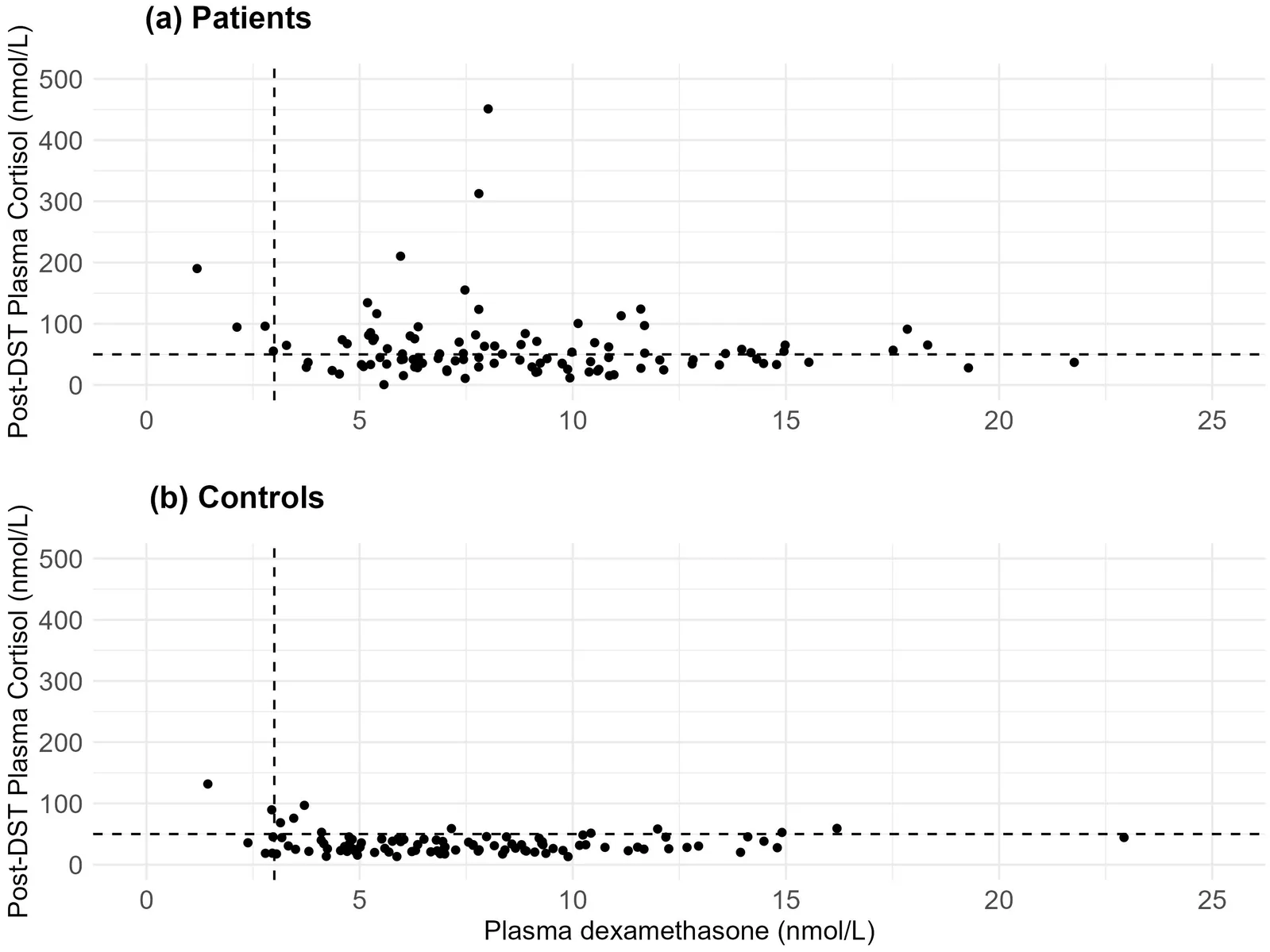
Relationship between liquid chromatography–tandem mass spectrometry (LC–MS/MS) plasma dexamethasone and cortisol following a 1 mg overnight dexamethasone suppression test (DST) in patients with adrenal incidentalomas (a, *N* = 107) and the control group (b, *N* = 98). The horizontal line represents the post-DST plasma cortisol cut-off concentration of 50 nmol/L. The vertical line represents the 2.5^th^ percentile (3.0 nmol/L).

Dexamethasone concentrations were not associated with post-DST cortisol in either patients (*P* = 0.5) or controls (*P* = 0.9) (**Figures 2A, B**).

### Guideline-recommended versus control-derived cortisol cut-off concentrations

**Figure 3:**
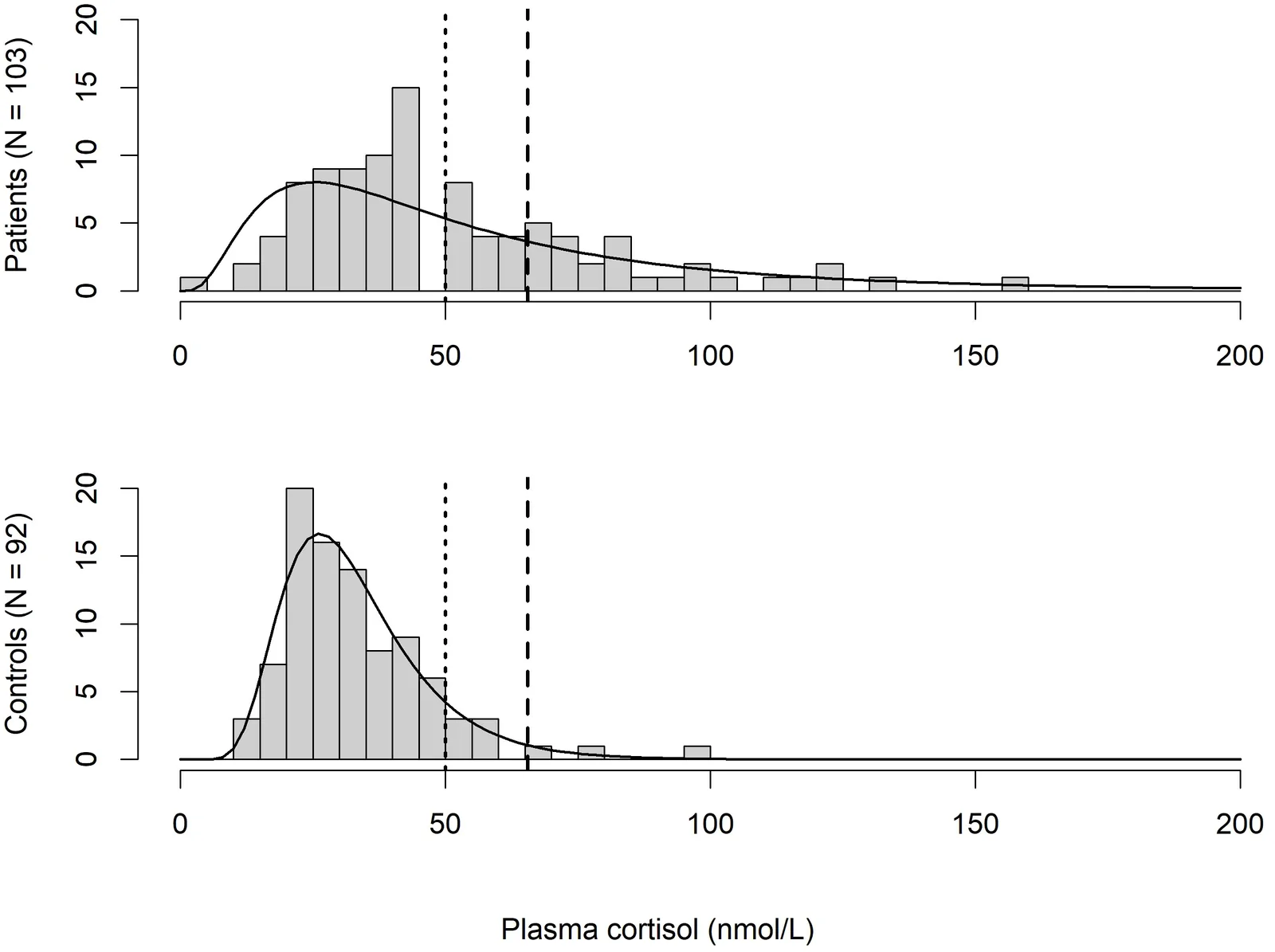
The distribution of liquid chromatography–tandem mass spectrometry (LC–MS/MS) plasma cortisol following a 1 mg overnight dexamethasone suppression test (DST) in patients with adrenal incidentalomas (“Patient group”, *N* = 103) (top figure) and the control group (“Control group”, *N* = 92) (bottom figure). Dotted line: currently recommended cut-off value (50 nmol/L); dashed line: 97.5^th^ percentile of the control group (66 nmol/L).

The guideline-recommended cut-off value of 50 nmol/L corresponded to the 90^th^ percentile of the post-DST cortisol distribution in controls, with 9.8% of controls and 43.7% of patients exceeding this threshold (**Figure 3**).

The 97.5^th^ percentiles in the control group were 63 nmol/L for Elecsys^®^Cort II and 66 nmol/L for LC–MS/MS, respectively (**Table 3**). Using the control-derived LC–MS/MS cut-off value of 66 nmol/L, 3.3% of controls and 26.2% of patients exceeded this threshold.

**Table 3:** Classification of patients with adrenal incidentalomas (*N* = 103) and controls (*N* = 92) according to guideline-recommended and control-derived post-DST cortisol cut-off values.

Cut-off definition	Cut-off value, nmol/L*	Patients above the cut-off value, *N* (%)	Controls above the cut-off value, *N* (%)
Guideline-defined cortisol cut-off value	50	45 (43.7)	9 (9.8)
LC-MS/MS-derived cut-off values, mean [95% CI]			
95^th^ percentile	58.0 [50.6–66.0]	35 (34.0)	6 (6)
97.5^th^ percentile	65.5 [56.3–75.6]	27 (26.2)	3(3)
Elecsys^®^Cort II-derived cut-off values, mean [95% CI]			
95^th^ percentile	55.0 [48.4–62.2]	35 (34.0)	6 (6.5)
97.5^th^ percentile	62.5 [54.2–71.7]	28 (27.2)	4 (4.3)

CI; confidence interval, Elecsys^®^Cort II; second-generation Roche Elecsys cortisol assay, LC–MS/MS; liquid chromatography–tandem mass spectrometry.

Results are presented as post-DST plasma cortisol cut-off values [95% CI] and the number (N (%)) of participants with concentrations above each cut-off value.

*Assay-specific 95^th^ and 97.5^th^ percentiles were estimated from fitted log-normal distributions in the control population with post-DST dexamethasone concentrations ≥3.0 nmol/L (N = 92). The guideline-recommended post-DST cortisol cut-off value was 50 nmol/L.

### Sensitivity analysis according to comorbidity burden in controls

In a sensitivity analysis excluding the 14 controls with ≥2 comorbidities, the 97.5^th^ percentile for post-DST plasma cortisol was 66.5 nmol/L (95% CI: 56.8–77.5), compared with 65.5 nmol/L (95% CI: 56.3–75.6) in the primary control group (*N* = 92).

### Pre-DST ACTH concentrations

Median pre-DST ACTH was lower in patients with post-DST cortisol >50 nmol/L than in those with ≤50 nmol/L (median (IQR): 2 (1–4) vs 3 (2–5) pmol/L; *P* < 0.001) and was lower in patients than in controls (**Table 1**; *P* < 0.001). In patients, lower pre-DST ACTH was associated with higher post-DST plasma cortisol (β = –0.27, *P* < 0.001), whereas no association was observed in controls (*P* = 0.4).

**Figure 4:**
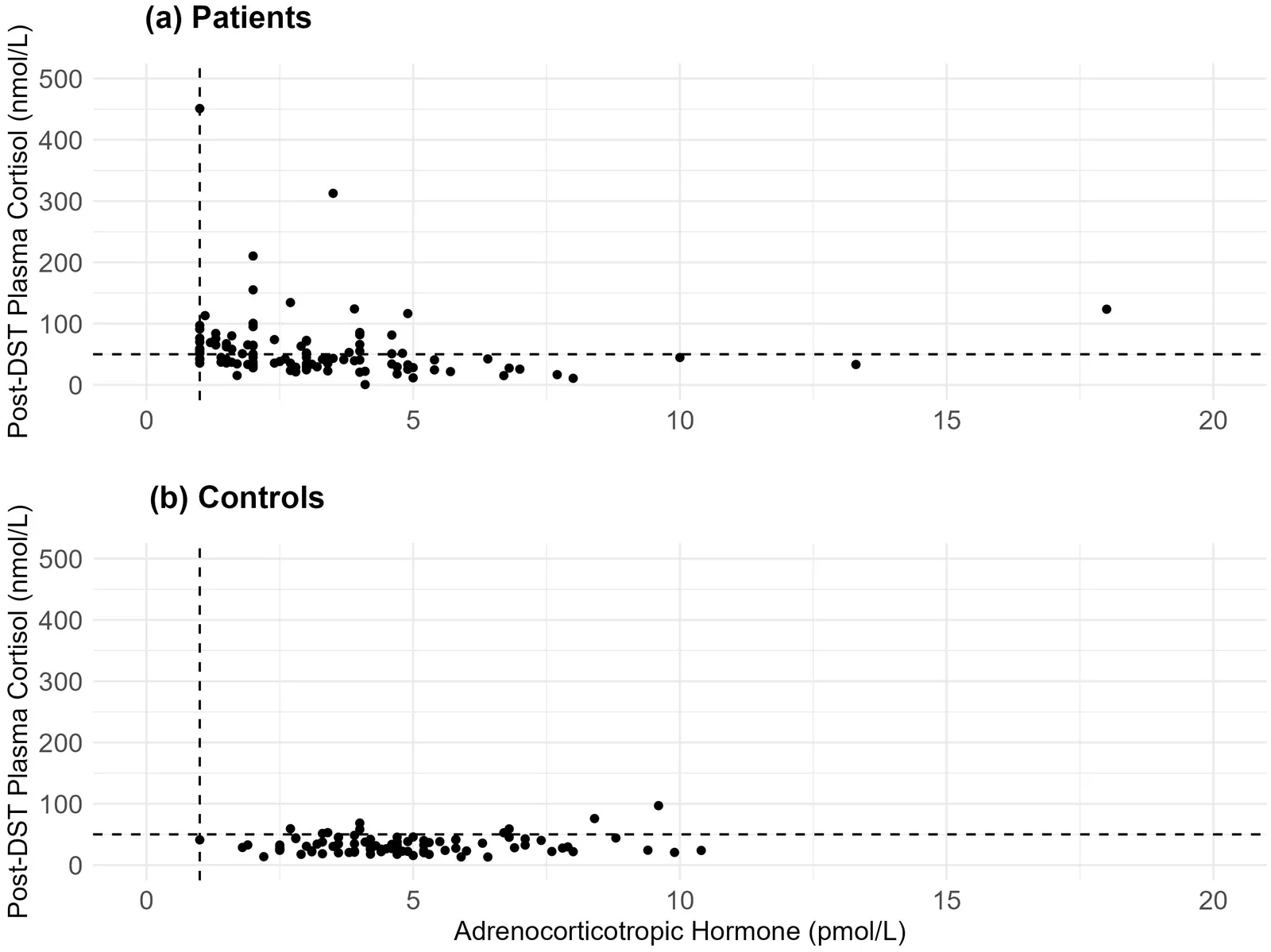
Relationship between immunoassay-measured ACTH (pre-DST) and plasma cortisol measured by liquid chromatography–tandem mass spectrometry (LC–MS/MS) after a 1 mg overnight dexamethasone suppression test (DST) in patients with adrenal incidentalomas **(a)**, *N* = 103) and the control group **(b)**, *N* = 92). The horizontal line indicates the post-DST plasma cortisol cut-off concentration of 50 nmol/L. The vertical line indicates the lower limit of quantification (LLOQ <1 pmol/L).

Ten patients (10%) had pre-DST ACTH concentrations below the LLOQ (<1 pmol/L), including one of the two patients diagnosed with Cushing’s syndrome; the second had a low-normal pre-DST ACTH concentration of 2 pmol/L. Among patients with ACTH below the LLOQ, three (30%) had a normal DST response (<50 nmol/L) (**Figure 4A**). One control (1%) had pre-DST ACTH below the LLOQ and a normal DST response (**Figure 4B**).

Pre-DST ACTH showed poor discrimination for post-DST cortisol >50 nmol/L (ROC AUC 0.69; 95% CI 0.60–0.80) and >66 nmol/L (ROC AUC 0.63; 95% CI 0.51–0.76).

### Cortisol-related comorbidities

After Bonferroni correction, higher post-DST cortisol concentrations were not significantly associated with diabetes (OR = 1.2, 95% CI: 0.5–2.9, *P* = 1.0), osteoporosis (OR = 1.3, 95% CI: 0.4–4.6, *P* = 1.0), hypertension (OR = 2.4, 95% CI: 1.1–5.2, *P* = 0.1), or dyslipidaemia (OR = 1.3, 95% CI: 0.7–2.3, *P* = 1.0). Exclusion of the two patients with Cushing’s syndrome did not change the overall findings (data not shown). Neither pre-DST ACTH concentrations nor the post-DST cortisol–pre-DST ACTH interaction was significantly associated with any of the comorbidities after Bonferroni correction (data not shown). Post-DST cortisol and pre-DST ACTH showed poor discriminatory ability for diabetes, hypertension, dyslipidaemia, and osteoporosis (all ROC AUCs 0.5–0.7).

Linear regression analyses showed that higher post-DST cortisol concentrations were associated with lower BMI (β = –1.7, *P* = 0.02) and lower waist circumference (β = –4.3, *P* = 0.02) after adjustment for sex and age; however, neither association remained statistically significant after Bonferroni correction. CBG concentrations were not significantly associated with BMI or waist circumference (data not shown). No significant associations were observed between post-DST cortisol and WHR or between age and post-DST cortisol (data not shown).

### Performance of the Elecsys^®^Cort II assay

**Figure 5:**
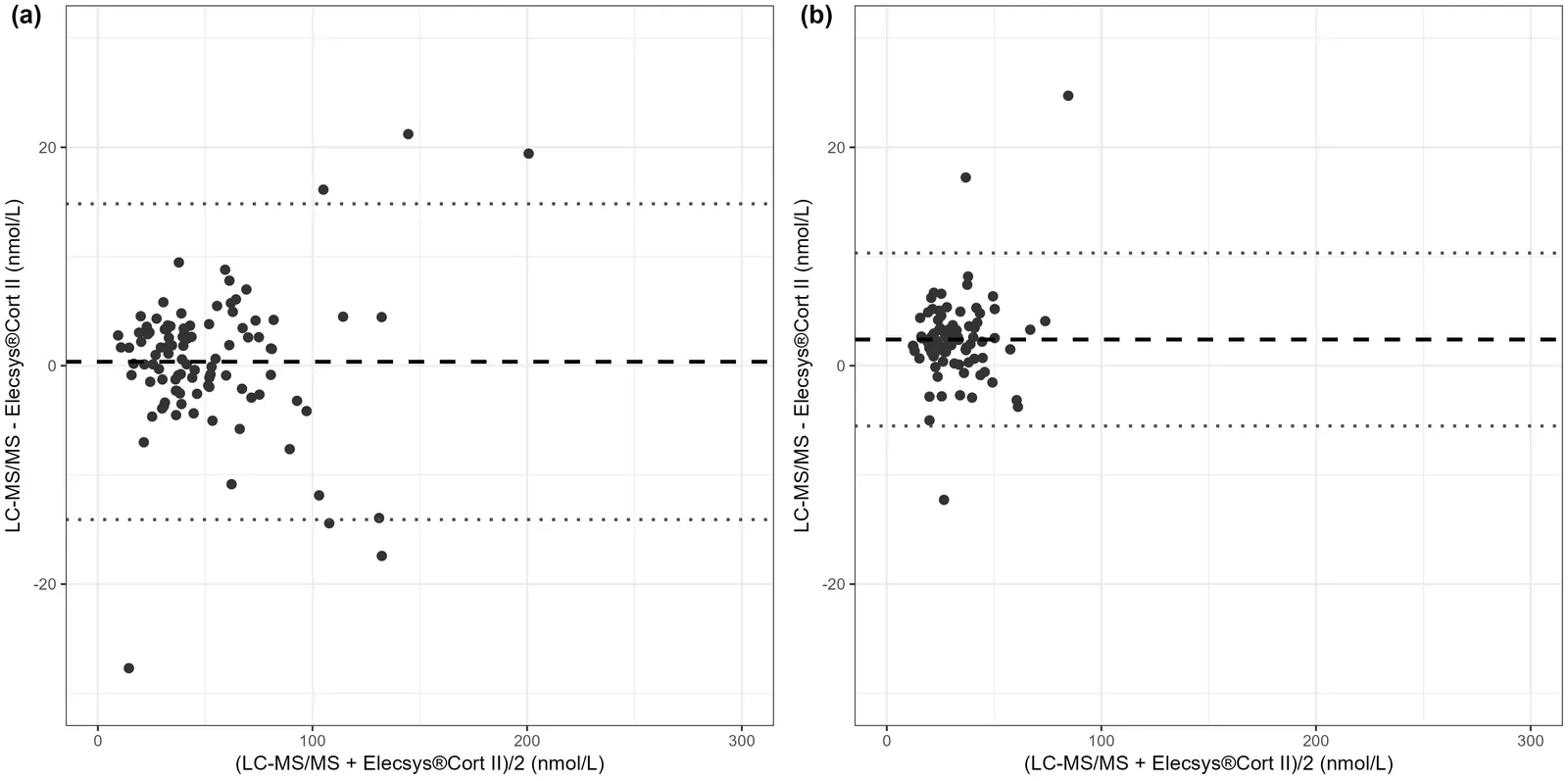
Agreement between the second-generation Roche Elecsys cortisol assay (Elecsys^®^Cort II) and liquid chromatography–tandem mass spectrometry (LC–MS/MS) for plasma cortisol following a 1 mg overnight dexamethasone suppression test (DST), assessed using Bland–Altman plots in patients with adrenal incidentalomas [N = 103 **(a)**] and controls [N = 92 **(b)**]. The dashed line represents the mean bias of Elecsys^®^Cort II relative to LC–MS/MS (a: 0.4 nmol/L; b: 2.4 nmol/L), and the dotted lines represent the 95% limits of agreement (a: –14.1 to 14.8 nmol/L; b: –5.5 to 10.3 nmol/L).

**Figure 6:**
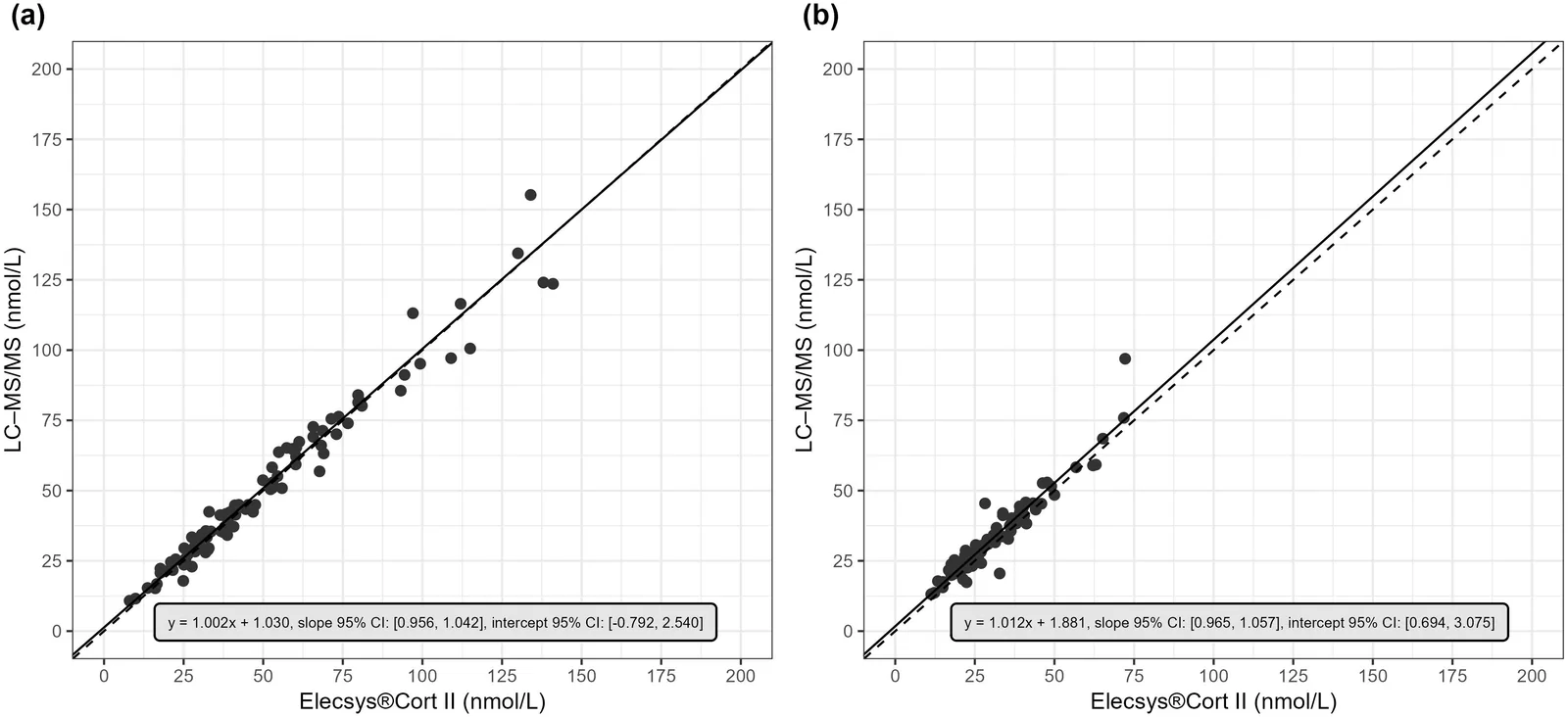
Agreement between the second-generation Roche Elecsys cortisol assay (Elecsys^®^Cort II) and liquid chromatography–tandem mass spectrometry (LC–MS/MS) for plasma cortisol following a 1 mg overnight dexamethasone suppression test (DST), assessed using Passing–Bablok regression in patients with adrenal incidentalomas [*N* = 103, **(a)**] and controls [*N* = 92, **(b)**] The dashed line represents the identity (x=y). The solid line represents the Passing–Bablok fit summarised in the grey box of each plot.

Post-DST cortisol concentrations measured by Elecsys^®^Cort II and LC–MS/MS, respectively, showed close agreement in both patients and controls (**Figures 5**, **6**).

The Bland–Altman analysis in patients showed a mean bias of 0.4 nmol/L [95% LoA: –14.1 to 14.8 nmol/L] (**Figure 5A**). For controls, the mean bias was 2.4 nmol/L [95% LoA: –5.5 to 10.3 nmol/L] (**Figure 5B**).

The Passing–Bablok regression analysis in the patient group showed an intercept of 1.03 nmol/L [95% CI: –0.79 to 2.54] and slope of 1.00 [95% CI: 0.96 to 1.04] (**Figure 6A**). In the control group, the intercept was 1.88 nmol/L [95% CI: 0.69 to 3.08], and the slope was 1.01 [95% CI: 0.96 to 1.06], (**Figure 6B**).

Sex did not influence the results of the Bland–Altman analysis or the Passing–Bablok regression analysis in either patients or controls (data not shown).

## Discussion

In the present study, we evaluated the recommended post-DST cortisol cut-off concentration for hypercortisolism—which in patients with adrenal incidentalomas predominantly represents MACS—using an age- and sex-matched control group. Measurement of post-DST dexamethasone allowed the identification and exclusion of participants with potentially inadequate dexamethasone exposure, thereby reducing the risk of interpreting insufficient cortisol suppression as hypercortisolism. In the resulting control group, the currently recommended cut-off value of 50 nmol/L corresponded to approximately the 90^th^ percentile, whereas the 97.5^th^ percentile was 66 nmol/L for LC–MS/MS. Using the cut-off value of 50 nmol/L, 44% of patients with adrenal incidentalomas were classified as having hypercortisolism compared with 26% using the 66 nmol/L threshold.

However, percentile-based cut-off values do not necessarily determine the clinically relevant threshold at which cortisol excess leads to adverse health outcomes (14).

The optimal post-DST cortisol threshold for defining MACS remains a matter of debate because of the absence of a gold standard and heterogeneity in study methods and MACS definitions (2, 15–18). In a meta-analysis (2), the currently recommended cut-off value of 50 nmol/L was associated with an increased prevalence of cardiometabolic comorbidities and increased mortality, although the evaluated thresholds showed poor discriminatory ability for these outcomes. Two large retrospective studies (19, 20) reported increased mortality at post-DST cortisol concentrations of approximately 80 nmol/L, whereas a cross-sectional multicentre study (21) observed increased cardiovascular morbidity only at concentrations above 138 nmol/L. Emerging evidence that patients with MACS may benefit from adrenalectomy further supports the clinical relevance of identifying these patients. However, increased mortality has also been reported in patients with non-functioning adenomas, suggesting that adverse outcomes may not be attributable solely to cortisol excess, although this remains controversial (4, 22).

Thus, although increasing post-DST cortisol concentrations have been associated with adverse outcomes (19–21, 23), the clinically relevant threshold remains uncertain. These findings support the need for outcome-based thresholds that better identify patients at risk of adverse outcomes or likely to benefit from treatment.

In the present study, higher post-DST cortisol concentrations were not significantly associated with diabetes, dyslipidaemia, hypertension, or osteoporosis after correction for multiple testing and showed poor discriminatory ability for these comorbidities, although a type II error cannot be excluded.

Pre-DST ACTH was inversely associated with post-DST plasma cortisol in patients; however, its discriminatory ability was poor, and 30% of patients with ACTH below the LLOQ had a normal DST response. Furthermore, neither pre-DST ACTH nor the interaction between post-DST cortisol and pre-DST ACTH was associated with cortisol-related comorbidities after correction for multiple testing.

These findings support the limited value of pre-DST ACTH as a screening marker for MACS (24), potentially because of its pulsatile secretion. Accordingly, current guidelines recommend ACTH primarily to establish ACTH independence in MACS (1). Alternative approaches, including post-DST ACTH and the basal cortisol-to-ACTH ratio, have been proposed for assessing cortisol excess, although evidence remains limited (5, 25, 26).

Simultaneous measurement of post-DST dexamethasone and cortisol has been suggested to improve DST performance, as low dexamethasone concentrations together with insufficient cortisol suppression may reflect poor compliance or rapid dexamethasone metabolism rather than autonomous cortisol secretion (8). In the present study, the 2.5^th^ percentile for post-DST dexamethasone was approximately 3.0 nmol/L, consistent with previously reported thresholds of 2.4 and 3.3 nmol/L (6–8). Six percent of controls and 4% of patients had dexamethasone concentrations below this threshold.

Notably, all four patients with dexamethasone concentrations <3.0 nmol/L had post-DST cortisol concentrations >50 nmol/L. Among controls with dexamethasone concentrations <3.0 nmol/L, two had post-DST cortisol >50 nmol/L. One underwent a repeat DST with adequate dexamethasone exposure and subsequently suppressed cortisol to 21 nmol/L. These findings suggest that inadequate dexamethasone exposure may contribute to apparent cortisol excess in some individuals. However, dexamethasone concentrations were not associated with post-DST cortisol in either patients or controls.

Importantly, the 3.0 nmol/L threshold and previously proposed thresholds (6–8) have largely been derived from dexamethasone distributions and do not necessarily define the concentration below which dexamethasone exposure becomes insufficient to suppress the hypothalamic-pituitary-adrenal (HPA) axis. Accordingly, the minimum dexamethasone concentration required for a valid DST remains uncertain. As in the present study, Klahr et al. (27) found no association between dexamethasone and cortisol concentrations and suggested that any quantifiable dexamethasone concentration may be sufficient for DST interpretation. Given the low frequency of inadequate dexamethasone exposure, selective measurement may represent an alternative to routine measurement in patients, as previously suggested (27). Future studies are needed to establish both the dexamethasone concentrations required for adequate suppression of the HPA axis and the optimal strategy for dexamethasone measurement in the clinical evaluation.

We observed an inverse association between post-DST cortisol concentrations and BMI and waist circumference, although these associations did not remain statistically significant after correction for multiple testing. Ueland et al. (8) also reported this inverse association, possibly reflecting lower CBG concentrations in individuals with obesity (28). However, CBG concentrations were not associated with BMI or waist circumference in the present study. Previous studies have reported an association between higher cortisol concentrations and increasing BMI (29, 30) or visceral fat (31, 32), while others have found no association (33–35).

We found no association between age and post-DST cortisol. Previous studies have reported conflicting results, ranging from no association (30, 36) to reduced cortisol suppression following DST in older individuals (35, 37–39).

This study evaluated the agreement between post-DST plasma cortisol measured by Elecsys^®^Cort II- and LC–MS/MS. The two methods showed close agreement with minimal bias, and regression analyses confirmed the absence of systematic or proportional differences in either patients or controls.

Previous cortisol immunoassays generally performed poorly compared with LC–MS/MS (10, 12, 40), but modern immunoassays using highly specific monoclonal antibodies and mass spectrometry-traceable calibration have demonstrated substantially improved accuracy compared with first-generation assays (40–42). Two previous studies also reported close agreement between Elecsys^®^Cort II and LC–MS/MS (10, 11).

Sex did not influence Elecsys^®^Cort II results, consistent with the findings of Atkins et al. (10) but in contrast to those of Hawley et al. (40).

The present study has limitations. The control group was smaller than recommended for establishing reference intervals (*N* = 120) (43). Furthermore, the study was not powered to assess associations between post-DST cortisol and cortisol-related comorbidities, and a type II error cannot be excluded. In addition, adrenal incidentalomas in the control group cannot be ruled out, as abdominal imaging was not performed in this group.

Imaging of apparently healthy controls was not considered ethically justified because of radiation exposure and the risk of incidental findings requiring further investigation. However, exclusion of controls with ≥2 comorbidities, who have been reported to have a higher prevalence of adrenal incidentalomas (44), had minimal influence on the control-derived threshold. Moreover, selecting controls from an apparently healthy population was considered more appropriate than recruiting individuals who had undergone abdominal imaging for clinical indications, as the latter may constitute a selected population.

This study also has strengths. The prospective inclusion of patients with adrenal incidentalomas and age- and sex-matched controls undergoing a standardised DST allowed a direct comparison of post-DST cortisol distributions between the two groups. Measurement of post-DST dexamethasone enabled restriction of analyses to participants with dexamethasone concentrations ≥3.0 nmol/L, reducing the influence of potentially insufficient dexamethasone exposure on the control-derived threshold.

In summary, the guideline-recommended cut-off value of 50 nmol/L corresponded to the 90^th^ percentile in controls, whereas the 97.5^th^ percentile was 66 nmol/L. Among patients with adrenal incidentalomas, 44% exceeded the guideline-recommended threshold compared with 26% exceeding the control-derived threshold. Post-DST dexamethasone concentrations were not associated with post-DST cortisol, highlighting the need to establish the dexamethasone concentration required for adequate suppression of the HPA axis. Pre-DST ACTH showed limited discriminatory ability for post-DST cortisol >50 nmol/L, and post-DST cortisol was not significantly associated with common cortisol-related comorbidities.

In conclusion, these findings suggest that the currently recommended post-DST cortisol cut-off value may be too low and that higher thresholds warrant further evaluation. However, a control-derived 97.5^th^ percentile does not necessarily define the clinically relevant threshold for cortisol excess, emphasising the need for an outcome-based definition of hypercortisolism (predominantly MACS) in patients with adrenal incidentalomas.

## Data Availability

The datasets presented in this article are not readily available because the data of this study are not publicly available due to ethical restrictions. Anonymised data are available from the corresponding author on reasonable request. Requests to access the datasets should be directed to marianne.christina.klose.01@regionh.dk.
