# Supplementary material for: Plasma cortisol cut-off concentrations using the dexamethasone suppression test in patients with adrenal incidentalomas
Source: Front Endocrinol (Lausanne). 2026 Sep 4;17:1872524. doi: 10.3389/fendo.2026.1872524 (PMC13585637; doi:10.3389/fendo.2026.1872524)
Supplement: Supplementary file 1 [file Table1.docx]

| Supplemental Table S1. Biochemical characteristics of controls with Elecsys®Cort II plasma cortisol concentrations >50 nmol/L following the 1 mg overnight dexamethasone suppression test | | | | | | |
| --- | --- | --- | --- | --- | --- | --- |
| No. | **First DST** | | | | **Repeat DST** | |
|  | **Pre-DST ACTH (pmol/L)** | **Post-DST cortisol (nmol/L)** | **Dexamethasone**  **(nmol/L)** | **Post-DST cortisol (nmol/L)** | | **Dexamethasone (nmol/L)** |
| 1 | 2.1 | 412.8 | 4.7 | 106.0 | | - |
| 2 | 2.4 | 359.5 | <1.25* | 323.0 | | <1.25* |
| 3 | 2.8 | 131.8 | 1.4 | 21.3 | | 8.1 |
| 4 | 9.6 | 96.9 | 3.7 | 27.0 | | 3.5 |
| 5 | 5.2 | 89.6 | 2.9 | - | | - |
| 6 | 8.4 | 75.9 | 3.5 | 76.0 | | - |
| 7 | 4.0 | 68.5 | 3.1 | - | | - |
| 8 | 2.7 | 59.2 | 16.2 | 72.4 | | 13.1 |
| 9 | 6.8 | 58.9 | 7.1 | 42.8 | | 9.4 |
| 10 | 4.0 | 58.2 | 12.0 | - | | - |
| 11 | 3.4 | 52.9 | 4,1 | - | | - |
| 12 | 6.7 | 52.6 | 14.9 | - | | - |
| 13 | 3.3 | 51.5 | 10.4 | - | | - |
| Abbreviations: ACTH, adrenocorticotropic hormone; Dexa, dexamethasone; DST, 1 mg overnight dexamethasone suppression test; No., number.  Results are presented in descending order. The controls were offered a repeat DST based on the Elecsys®Cort II post-DST plasma cortisol measurements, the assay routinely used in The Department of Clinical Biochemistry, Rigshospitalet, Copenhagen University Hospital. Seven controls underwent a repeat test. ACTH lower limit of quantification (LLOQ) was <1 pmol/L (between 0700-1000 hours). Plasma dexamethasone concentrations were repeated in five controls.  *Below LLOQ | | | | | | |
